# Predictive value of preoperative [18F]FDG PET/CT imaging parameters for postoperative recurrence and metastasis in breast cancer patients

**DOI:** 10.3389/fonc.2026.1946005

**Published:** 2026-10-21

**Authors:** Nan Lin, Chao Ren, Zhe Wen

**Affiliations:** 1 Department of Nuclear Medicine, Beijing Shijitan Hospital, Capital Medical University, Beijing, China; 2 Department of Nuclear Medicine, Beijing Key Laboratory of Molecular Targeted Diagnosis and Therapy in Nuclear Medicine, Peking Union Medical College Hospital, Chinese Academy of Medical Science & Peking Union Medical College, Beijing, China

**Keywords:** breast cancer, computed tomography, metasis, positron emission tomography, predictive value, recurrence

## Abstract

**Purpose:**

To assess the predictive value of preoperative 2-deoxy-2-[¹^8^F]fluoro-D-glucose positron emission tomography/computed tomography ([18F]FDG PET/CT) imaging parameters in forecasting the recurrence and metastasis following surgery among individuals diagnosed with early-stage breast cancer.

**Methods:**

A retrospective analysis was performed on 106 female patients with early-stage breast cancer (clinical TNM (cTNM) stage I–II, T1–2, N0–1, M0), all of whom underwent surgical procedures at our institution between January 2021 and December 2023. These individuals were divided into two groups: a non-recurrence group (n=57) and an recurrence/metastasis group (n=49), determined by whether recurrence or metastasis was observed within 24 months postoperatively. Preoperative imaging was carried out using a Discovery 710 scanner, and metabolic parameters were measured. Histological markers were assessed via immunohistochemistry.

**Results:**

Significant differences were observed in lymphovascular invasion, Ki-67 expression, ER positivity, PR positivity, HER2 status, and TNBC subtype between the two groups. Among PET/CT parameters examined, it was observed that the recurrence/metastasis group exhibited significantly higher values for primary tumor (T) metabolic tumor volume (MTV), T total lesion glycolysis (TLG), axillary lymph nodes (N), maximum standardized uptake value (SUVmax), whole body (WB) MTV, and WB TLG. Multivariate logistic regression identified T MTV, T TLG, N SUVmax, WB MTV, and WB TLG as independent risk factors for recurrence and metastasis (all P<0.05). Receiver operating characteristic (ROC) analysis demonstrated that these parameters had good predictive performance, with combined ROC yielding an area under the curve of 0.805.

**Conclusion:**

Preoperative [18F]FDG PET/CT imaging parameters may be valuable predictors of postoperative recurrence and metastasis in early-stage breast cancer patients. These findings highlight the potential utility of metabolic imaging in guiding personalized treatment strategies.

## 1 Introduction

Breast cancer remains a leading malignancy globally, associated with substantial rates of illness and death, despite advancements in early detection and treatment (1). Early-stage breast cancer is often curable, but a subset of patients still experiences recurrence or metastasis after primary treatment (2). Accurate prediction of these adverse outcomes is crucial for optimizing therapeutic strategies and improving patient outcomes. Imaging modalities play a pivotal role in assessing tumor characteristics and predicting prognosis. Particularly, 2-deoxy-2-[^18^F]fluoro-D-glucose positron emission tomography.

computed tomography ([18F]FDG PET/CT) has become a valuable method due to its ability to measure metabolic activity, which correlates with tumor aggressiveness (3).

The utilization of [18F]FDG PET/CT in cancer diagnostics is well-established, providing comprehensive information on tumor metabolism, extent, and distribution (4). This imaging technique quantifies glucose uptake in tissues, reflecting the metabolic rate of malignant cells. Increased glucose metabolism is a hallmark of many cancers, including breast cancer, where it supports rapid proliferation and survival (5). Metrics obtained from PET/CT scans, including the standardized uptake value (SUV), metabolic tumor volume (MTV), and total lesion glycolysis (TLG), have shown correlations with tumor biology and clinical outcomes. Specifically, MTV and TLG integrate metabolic activity and tumor size, offering a more thorough assessment of tumor burden than SUV alone (5). These parameters have demonstrated prognostic value in various cancers, suggesting their potential utility in breast cancer management (6, 7).

Histological markers, including the proliferation marker Ki-67, estrogen receptor (ER), progesterone receptor (PR), and human epidermal growth factor receptor 2 (HER2), are commonly used to characterize breast tumors and guide treatment decisions. Ki-67 reflects cellular proliferation, with higher expression levels associated with more aggressive tumors (8). ER and PR status influence hormone therapy response, while HER2 positivity indicates eligibility for targeted therapies. Despite the prognostic value of these markers, they do not fully capture the complexity of tumor behavior, particularly in terms of metabolic activity and systemic spread (9). Consequently, integrating molecular and metabolic data may enhance predictive accuracy and provide a more holistic understanding of tumor biology.

The importance of preoperative imaging in breast cancer extends beyond initial diagnosis and staging. Preoperative PET/CT can identify distant metastases and assess primary tumor characteristics, which are critical for surgical planning and treatment strategy formulation. Moreover, preoperative imaging parameters may serve as biomarkers for predicting postoperative outcomes, such as recurrence and metastasis (10). Despite the increasing evidence highlighting the prognostic significance of PET/CT parameters, their application in routine clinical practice remains limited. Challenges include standardization of imaging protocols, variability in image interpretation, and the need for robust validation across diverse populations. Addressing these challenges requires collaborative efforts among researchers, clinicians, and imaging specialists to develop consensus guidelines and enhance the reproducibility of PET/CT-based assessments (11, 12).

The potential of preoperative [18F]FDG PET/CT imaging parameters to predict postoperative recurrence and metastasis in breast cancer patients represents an area of active investigation. The ability of these parameters to provide insights into tumor biology and guide therapeutic decisions underscores their clinical significance. This research aimed to enhance the expanding knowledge base by evaluating the predictive power of PET/CT parameters in the context of breast cancer recurrence and metastasis.

## 2 Materials and methods

### 2.1 Study subjects

Between January 2021 and December 2023, female patients diagnosed with early-stage breast cancer and treated surgically at our institution were followed up for 24 months via telephone and outpatient visits. Excluding cases lost to follow-up and deaths, this study included 106 patients. Inclusion criteria were as follows: (a) meeting the diagnostic criteria for primary breast cancer (13) and having a single tumor; (b) clinical TNM stage I-II (T1–2, N0–1, M0) according to the AJCC Cancer Staging Manual, 8th edition (14), corresponding to early-stage breast cancer; (c) undergoing [18F]FDG PET/CT imaging within one week before surgery; and (d) having complete and reliable medical records and imaging data. Exclusion criteria included: (a) prior history of breast cancer or other malignant tumors; (b) poor image quality that precluded accurate assessment; (c) presence of severe underlying diseases or active infections that could affect the study results; and (d) receiving neoadjuvant chemotherapy, radiotherapy, or targeted therapy prior to surgery.

### 2.2 Study group

Based on whether recurrence or metastasis occurred within 24 months postoperatively, the 106 patients were categorized into a non-recurrence group (n = 57) and an recurrence/metastasis group (n = 49). The non-recurrence group was defined as patients who showed no evidence of recurrence or metastasis within 24 months after surgery. The recurrence/metastasis group was defined as patients who experienced confirmed recurrence or metastasis within 24 months after surgery, including 19 cases of lymph node metastasis, 6 cases of chest wall recurrence, 3 cases of pleural metastasis, 15 cases of bone metastasis, and 6 cases of lung metastasis. For patients with multiple sites of involvement, the site of first detection or the clinically dominant site was recorded as the primary site. Each patient was counted once. The diagnostic criteria for recurrence and metastatic lesions (15) were: (1) postoperative reoperation or pathological confirmation after biopsy; (2) imaging follow-up of metastatic lesions based on two or more imaging results: (a) during follow-up, existing lesions increased in size or new lesions appeared, and were diagnosed as metastatic lesions by ultrasound, CT, magnetic resonance imaging (MRI), or single-photon emission computed tomography (SPECT); (b) after anti-tumor treatment, imaging showed significant reduction or disappearance of the original metastatic lesion, consistent with clinical improvement, which was also considered indicative of metastasis. This retrospective investigation received approval from our institutional review board, and accessing medical records and imaging data did not require patient authorization or informed consent.

### 2.3 Imaging methods

All patients received preoperative PET/CT imaging with a Discovery 710 scanner (GE Healthcare, Milwaukee, WI, USA) (**Figures 1**, **2**). Prior to the scan, patients were instructed to fast for 6 to 8 hours, with fasting blood glucose levels remaining below 7.0 mmol/L. A dose of 4.44 to 5.18 MBq/kg of [18F]FDG (radiochemical purity >95%, China Institute of Atomic Energy) was administered intravenously. Patients rested for 50 to 60 minutes before imaging. A low-dose 16-slice helical CT scan was performed first with these settings: slice thickness of 3.75 mm, tube voltage of 120 kV, tube current of 40–100 mA automatically adjusted to tissue depth, pitch of 1.0 mm, and a scan range from head to thigh for anatomical localization and attenuation correction of PET images. This was followed by PET scanning with the same scan range as the CT, with each bed position scanned for approximately 2 to 3 minutes in 3D acquisition mode to obtain attenuation-corrected images. After scanning, PET/CT images were reconstructed using an iterative reconstruction algorithm (OSEM) (2 iterations, 8 subsets, 128×128 matrix, 5-mm Gaussian filter). A GE Advantage Workstation version 4.6 with 3D volume viewer software was utilized for quantitative analysis to derive different metabolic parameters of the primary tumor (T) and axillary lymph nodes (N): SUV (SUVmax and SUVmean), MTV, and TLG. The sums of these parameters were calculated for the whole body (WB), encompassing SUVmax, SUVmean, MTV, and TLG.

**Figure 1:**
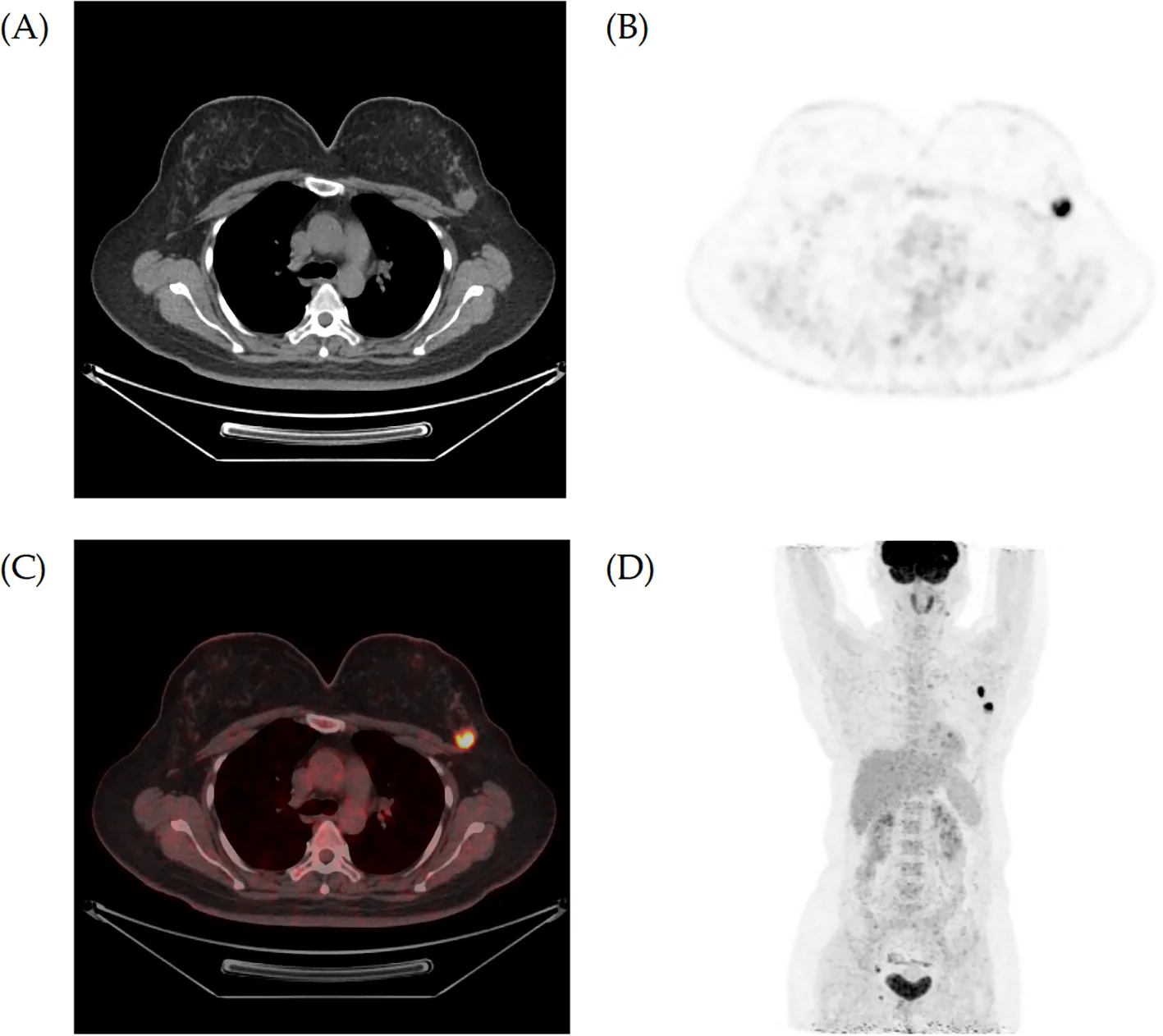
[18F]FDG PET/CT images of a typical patient in recurrence/metastasis group. **(A)** CT anatomical image; **(B)** PET metabolic image; **(C)** CT-PET fusion image; **(D)** Whole-body PET image.

**Figure 2:**
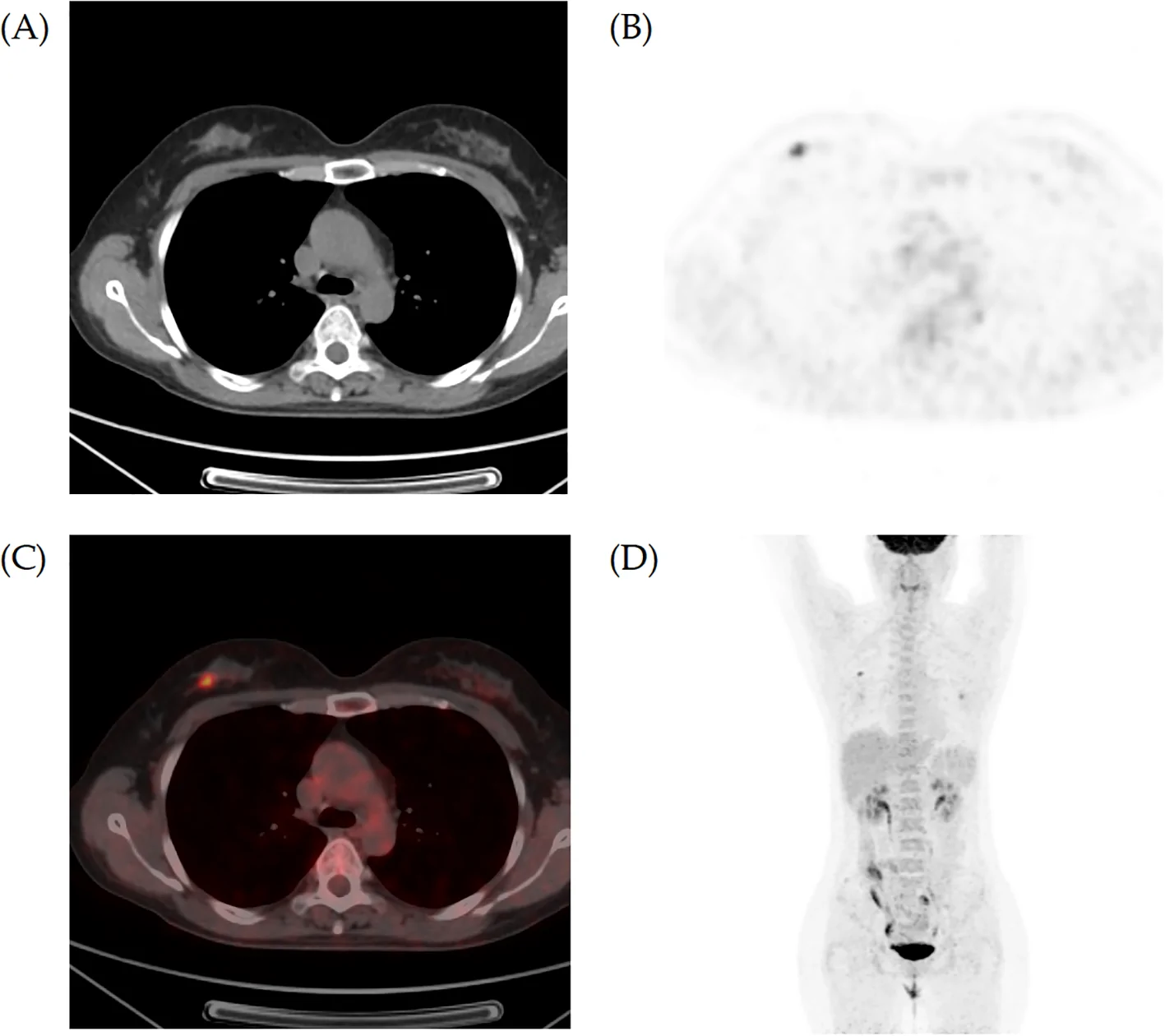
[18F]FDG PET/CT images of a typical patient in non-recurrence group. **(A)** CT anatomical image; **(B)** PET metabolic image; **(C)** CT-PET fusion image; **(D)** Whole-body PET image.

For axillary nodal assessment, the ipsilateral axillary basin was systematically evaluated by two experienced nuclear medicine physicians. For patients with a visually identifiable FDG-avid axillary lymph node, a 3D volume of interest (VOI) was placed over the node with the highest FDG uptake, and N SUVmax, N SUVmean, N MTV, and N TLG were recorded. For patients without an identifiable FDG-avid axillary lymph node, a standardized circular ROI was placed over the axillary nodal basin at the level of the axillary vessels, and the SUVmax within the ROI was recorded as N SUVmax; N MTV and N TLG were recorded as 0 in these cases. This approach ensured that all patients, including those with stage I disease and no detectable abnormal lymph nodes, had complete nodal PET/CT parameter data for analysis.

### 2.4 Histopathology

Postoperatively, the expression status of Ki-67, ER, PR, and HER2 in surgical specimens was detected through immunohistochemistry (IHC). Detection utilized rabbit anti-human Ki-67 monoclonal antibody kits (ZM-0166, Beijing Zhongshan Golden Bridge Biotechnology Co., Ltd., China), ER detection kits (790-4325, Ventana, USA), PR detection kits (RM-9102-S, Beijing Zhongshan Golden Bridge Biotechnology Co., Ltd., China), and HER2 detection kits (790-4493, Ventana, USA). The instrument used was a fully automated immunohistochemical staining machine (Bond-III, Leica, Germany or BenchMark Ultra, Ventana, USA). ER and PR positivity were determined using the Allred score (16), which combines the proportion score and intensity score of positive-stained tumor cells, ranging from 0 to 8. HER2 staining intensity scores were 0, 1+, 2+, or 3+ (17). Tumors with a score of 3 were considered HER2-positive, while tumors with scores of 0 or 1 were considered HER2-negative. Tumors scoring 2 underwent fluorescence *in situ* hybridization (FISH) using a fluorescence *in situ* hybridization instrument (TDH 500, Hangzhou Aosheng Instrument Co., Ltd., China) to ascertain the HER2 status. A tumor was classified as HER2-positive if the HER2/CEP17 ratio (the proportion of HER2 signals to chromosome 17 probe signals) exceeded 2.0. Triple-negative breast cancer (TNBC) was defined as ER-negative (Allred score <3), PR-negative (Allred score <3), and HER2-negative (IHC 0 or 1+, or IHC 2+ with HER2/CEP17 ratio ≤2.0 on FISH) (18).

Additionally, lymphovascular invasion (LVI) was assessed as part of routine postoperative histopathological examination of the surgical specimen. Formalin-fixed, paraffin-embedded tissue sections were stained with hematoxylin and eosin (H&E) and examined by board-certified pathologists. LVI was defined as the presence of tumor cell emboli within lymphatic or blood vessel spaces lined by endothelial cells. LVI status was recorded as positive or negative based on the final pathology report. Immunohistochemical staining for endothelial markers (e.g., D2-40, CD31, or CD34) was not routinely performed for LVI assessment; such staining was used only at the pathologist’s discretion in selected equivocal cases, if necessary. No central pathology review was performed.

### 2.5 Statistical methods

This research utilized SPSS 29.0 statistical software (SPSS Inc., USA) for data analysis. Continuous data were expressed as mean ± standard deviation (M ± SD) and were compared between groups utilizing independent samples t-tests. Categorical data were presented as frequencies and percentages [n(%)] and were compared between groups with the χ² tests. Statistical significance was established with a P-value less than 0.05. Multivariate logistic regression analyses were carried out, with “recurrence or metastasis within 24 months postoperatively” as the dependent variable (no recurrence = 0, recurrence = 1), to screen for independent risk factors affecting postoperative recurrence and metastasis in early-stage breast cancer patients. Finally, receiver operating characteristic (ROC) curve analysis was carried out to assess the predictive capability of the identified independent risk factors for postoperative recurrence and metastasis in patients with early-stage breast cancer.

Candidate variables included PET/CT parameters (T MTV, T TLG, N SUVmax, WB MTV, WB TLG) and clinicopathological variables (LVI, pathological N stage [pN]). Because MTV and TLG are mathematically related and strongly correlated, and because the number of events limited the number of variables that could be reliably estimated, we constructed separate multivariable logistic regression models. Each model included one PET parameter and two fixed clinically established covariates: LVI and pN. Molecular markers (ER, PR, HER2, Ki-67) were not included in the multivariable models because they are components of molecular subtype, are strongly associated with PET parameters, and would increase the risk of overfitting. This approach avoided multicollinearity among PET parameters and ensured an adequate events-per-variable ratio. Pearson correlation coefficients were calculated among PET parameters. Multicollinearity was assessed using variance inflation factor (VIF) and tolerance values. VIF > 10 and tolerance < 0.1 were considered indicative of serious multicollinearity. VIF was calculated for a model containing all five PET parameters simultaneously and for each separate multivariable model.

Subgroup analyses were performed to evaluate the consistency of the prognostic value of PET/CT metabolic parameters across histologic type (invasive ductal carcinoma [IDC] vs. other types) and type of surgery (breast-conserving surgery [BCS] vs. mastectomy) strata. Multivariate logistic regression was performed separately in each subgroup. The interaction between PET/CT parameters and subgroup variables was assessed by including an interaction term in the regression model, and the likelihood ratio test was used to evaluate the significance of the interaction. All subgroup analyses were exploratory and hypothesis-generating.

## 3 Results

### 3.1 Demographic data

The comparison of demographic data between the non-recurrence group (n=57) and the recurrence/metastasis group (n=49) showed no significant differences in any of the baseline characteristics (**Table 1**). No significant differences were observed in age (P = 0.806), body mass index (BMI) (P = 0.562), ethnicity (P = 0.836), drinking history (P = 0.535), smoking history (P = 0.372), hypertension (P = 0.795), diabetes (P = 0.551), family history of breast cancer (P = 0.397), or menstrual status (P = 0.462). These results suggest that the baseline demographic characteristics were comparable between the two groups, indicating that any observed outcomes are unlikely to be influenced by these factors.

**Table 1:** Comparison of demographic data between two groups.

Parameter	Non-recurrence group (n=57)	Recurrence/metastasis group (n=49)	t/χ^2^	P
Age (years)	47.86 ± 5.39	48.13 ± 5.64	0.247	0.806
BMI (kg/m^2^)	22.45 ± 1.03	22.57 ± 1.21	0.582	0.562
Ethnicity [n(%)]			0.043	0.836
Han	54 (94.74%)	45 (91.84%)		
Others	3 (5.26%)	4 (8.16%)		
Drinking history[n(%)]			0.384	0.535
Yes	12 (21.05%)	8 (16.33%)		
No	45 (78.95%)	41 (83.67%)		
Smoking history[n(%)]			0.798	0.372
Yes	5 (8.77%)	7 (14.29%)		
No	52 (91.23%)	42 (85.71%)		
Hypertension [n(%)]			0.067	0.795
Yes	15 (26.32%)	14 (28.57%)		
No	42 (73.68%)	35 (71.43%)		
Diabetes [n(%)]			0.355	0.551
Yes	7 (12.28%)	8 (16.33%)		
No	50 (87.72%)	41 (83.67%)		
Family history of breast cancer [n(%)]			0.717	0.397
Yes	9 (15.79%)	5 (10.20%)		
No	48 (84.21%)	44 (89.80%)		
Menstrual status [n(%)]			0.542	0.462
Premenopausal	32 (56.14%)	24 (48.98%)		
Postmenopausal	25 (43.86%)	25 (51.02%)		

BMI, body mass index.

### 3.2 Clinicopathological and surgical data

No significant differences were observed in TNM stage (P = 0.238), N stage (P = 0.237), histologic type (P = 0.729), tumor size (P = 0.280), or type of surgery (P = 0.596) (**Table 2**). However, a notable difference was found in LVI (P = 0.039), with 8 (14.04%) patients showing positive results in the non-recurrence group compared to 15 (30.61%) in the recurrence/metastasis group.

**Table 2:** Comparison of clinicopathological and surgical data between two groups.

Parameter	Non-recurrence group (n=57)	Recurrence/metastasis group (n=49)	t/χ^2^	P
TNM stage [n(%)]			1.395	0.238
Stage I	25 (43.86%)	16 (32.65%)		
Stage II	32 (56.14%)	33 (67.35%)		
N stage [n(%)]			1.401	0.237
pN0	40 (70.18%)	29 (59.18%)		
pN1	17 (29.82%)	20 (40.82%)		
Histologic type [n(%)]			0.120	0.729
IDC	45 (78.95%)	40 (81.63%)		
Other types	12 (21.05%)	9 (18.37%)		
Tumor size (cm)	3.32 ± 1.06	3.56 ± 1.18	1.085	0.280
LVI [n(%)]			4.262	0.039
Positive	8 (14.04%)	15 (30.61%)		
Negative	49 (85.96%)	34 (69.39%)		
Type of surgery [n(%)]			0.281	0.596
BCS	25 (43.86%)	19 (38.78%)		
Mastectomy	32 (56.14%)	30 (61.22%)		

TNM, tumor, node, metastasis; pN, pathological N stage; IDC, invasive ductal carcinoma; LVI, lymphovascular invasion; BCS, breast-conserving surgery.

### 3.3 Histological marker

Notable differences were noted in Ki-67 expression (P = 0.046), with 32 (56.14%) patients showing levels below 14% in the non-recurrence group compared to 18 (36.73%) in the recurrence/metastasis group, while 25 (43.86%) and 31 (63.27%) patients had levels of 14% or higher, respectively (**Table 3**). Estrogen receptor (ER) positivity was significantly different (P = 0.043), with 40 (70.18%) patients being positive in the non-recurrence group versus 25 (51.02%) in the recurrence/metastasis group, and 17 (29.82%) and 24 (48.98%) patients being negative, respectively. Progesterone receptor (PR) positivity also showed a significant difference (P = 0.039), with 37 (64.91%) patients were positive in the non-recurrence group compared to 22 (44.90%) in the recurrence/metastasis group, and 20 (35.09%) and 27 (55.10%) patients were negative, respectively. Additionally, HER2 positivity was significantly different (P = 0.046), with 14 (24.56%) patients being positive in the non-recurrence group compared to 21 (42.86%) in the recurrence/metastasis group, and 43 (75.44%) and 28 (57.14%) patients being negative, respectively. Finally, when receptor status was integrated into a descriptive molecular subtype analysis, TNBC (ER−/PR−/HER2−) was identified in 4 of 57 patients (7.02%) in the non-recurrence group and 11 of 49 patients (22.45%) in the recurrence/metastasis group, showing a statistically significant difference (χ^2^ = 3.973, P = 0.046). This finding is consistent with the well-established association between TNBC and a higher risk of recurrence and metastasis.

**Table 3:** Comparison of histological marker between two groups [n(%)].

Parameter	Non-recurrence group (n=57)	Recurrence/metastasis group (n=49)	χ^2^	P
Ki-67			3.982	0.046
<14	32 (56.14%)	18 (36.73%)		
≥14	25 (43.86%)	31 (63.27%)		
ER			4.076	0.043
Positive	40 (70.18%)	25 (51.02%)		
Negative	17 (29.82%)	24 (48.98%)		
PR			4.277	0.039
Positive	37 (64.91%)	22 (44.90%)		
Negative	20 (35.09%)	27 (55.10%)		
HER2			3.988	0.046
Positive	14 (24.56%)	21 (42.86%)		
Negative	43 (75.44%)	28 (57.14%)		
Molecular subtype			3.973	0.046
TNBC	4 (7.02%)	11 (22.45%)		
Non-TNBC	53 (92.98%)	38 (77.55%)		

Ki-67, cellular proliferation index; ER, estrogen receptors; PR, progesterone receptors; HER2, human epidermal growth factor receptor 2; TNBC, triple-negative breast cancer.

### 3.4 Preoperative [18F]FDG PET/CT imaging parameters

No significant differences were observed in T SUVmax (P = 0.077) or T SUVmean (P = 0.091) (**Figure 3**). Significant differences were observed in T MTV (18.27 ± 5.33 vs 21.95 ± 6.72, t=3.139, P = 0.002) and T TLG (86.84 ± 20.25 vs 100.47 ± 32.18, t=2.561, P = 0.012), with both metrics being higher in the recurrence/metastasis group.

**Figure 3:**
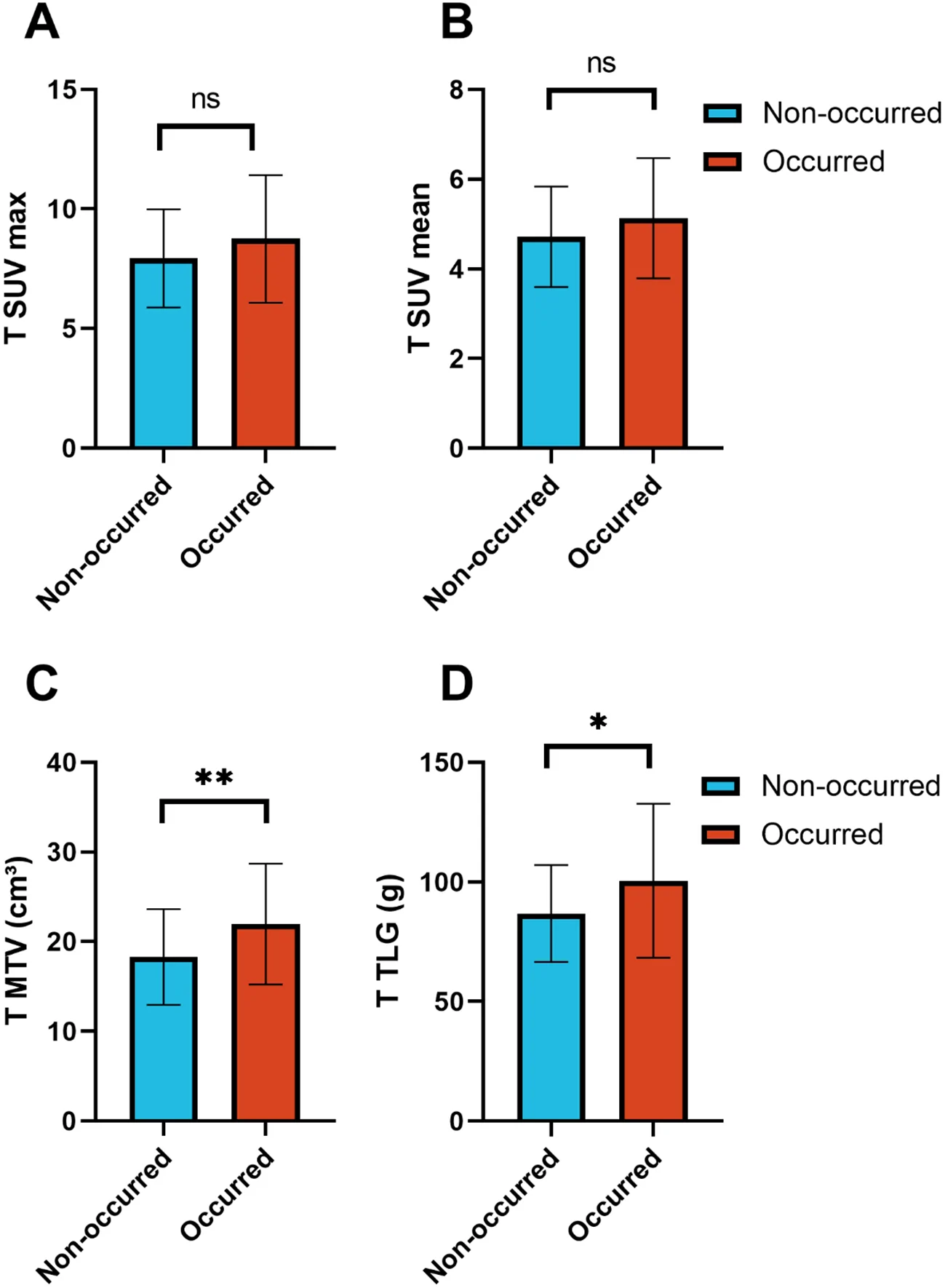
Comparison of preoperative [18F]FDG PET/CT imaging parameters of the primary tumor between two groups **(A)** T SUVmax; **(B)** T SUVmean; **(C)** T MTV; **(D)** T TLG. T, primary tumor; SUV, standardized uptake value; MTV, metabolic tumor volume; TLG, total lesion glycolysis. ns, no significant difference; *: P<0.05; **: P<0.01.

T, primary tumor; SUV, standardized uptake value; MTV, metabolic tumor volume; TLG, total lesion glycolysis. ns: no significant difference; *: P<0.05; **: P<0.01.

No significant differences were observed in N SUVmean (P = 0.156), N MTV (P = 0.058), or N TLG (P = 0.051) (**Figure 4**). However, a notable difference was found in N SUVmax (3.18 ± 0.95 vs 3.74 ± 1.18, t=2.724, P = 0.008), with higher values in the recurrence/metastasis group.

**Figure 4:**
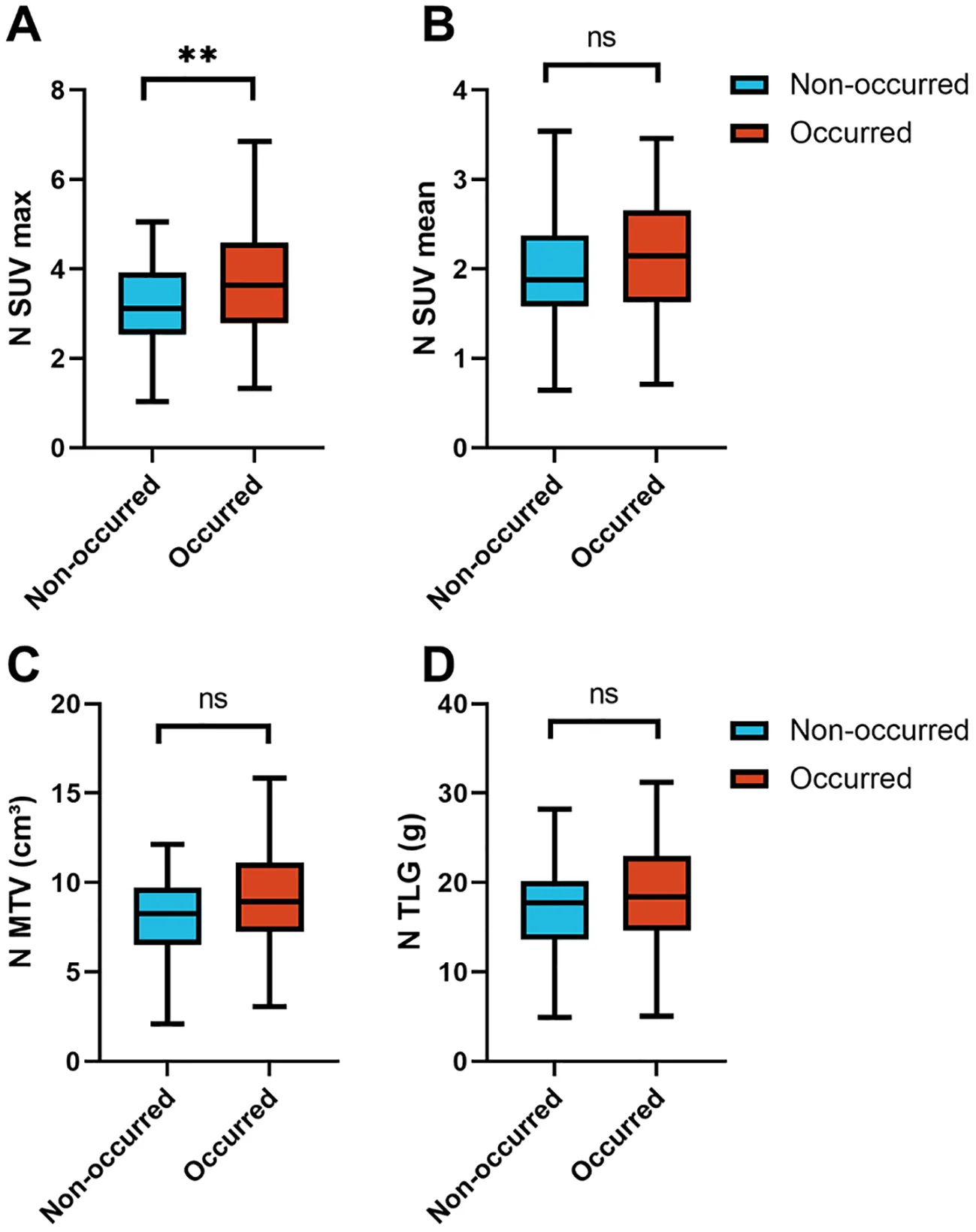
Comparison of preoperative [18F]FDG PET/CT imaging parameters of the axillary lymph nodes between two groups **(A)** N SUVmax; **(B)** N SUVmean; **(C)** N MTV; **(D)** N TLG. N, axillary lymph nodes; SUV, standardized uptake value; MTV, metabolic tumor volume; TLG, total lesion glycolysis. ns, no significant difference; **: P<0.01.

N, axillary lymph nodes; SUV, standardized uptake value; MTV, metabolic tumor volume; TLG, total lesion glycolysis. ns: no significant difference; **: P<0.01.

No significant differences were observed in WB SUVmax (P = 0.055) or WB SUVmean (P = 0.106) (**Table 4**). However, notable differences were observed in WB MTV (26.32 ± 7.83 vs 30.06 ± 8.14, t=2.409, P = 0.018) and WB TLG (103.58 ± 26.42 vs 119.29 ± 30.67, t=2.834, P = 0.006), with both metrics being higher in the recurrence/metastasis group.

**Table 4:** Comparison of preoperative [18F]FDG PET/CT imaging parameters of the whole body between two groups.

Parameter	Non-recurrence group (n=57)	Recurrence/metastasis group (n=49)	t	P
WB SUVmax	11.11 ± 2.97	12.49 ± 4.12	1.947	0.055
WB SUVmean	6.67 ± 1.64	7.25 ± 2.03	1.629	0.106
WB MTV (cm^3^)	26.32 ± 7.83	30.06 ± 8.14	2.409	0.018
WB TLG (g)	103.58 ± 26.42	119.29 ± 30.67	2.834	0.006

WB, whole body; SUV, standardized uptake value; MTV, metabolic tumor volume; TLG, total lesion glycolysis.

### 3.5 Multivariate logistic regression

Pearson correlation analysis revealed strong correlations among PET parameters: T MTV and T TLG (r = 0.892), WB MTV and WB TLG (r = 0.915), T MTV and WB MTV (r = 0.785), and T TLG and WB TLG (r = 0.803). When all five PET parameters were entered simultaneously into a single multivariable model, VIF values exceeded 10 for multiple parameters (T MTV VIF = 12.456; T TLG VIF = 15.782; WB MTV VIF = 11.234; WB TLG VIF = 14.567; N SUVmax VIF = 3.214), confirming serious multicollinearity. Therefore, five separate multivariable models were constructed, each containing one PET parameter plus LVI and pN. Within each separate model, VIF values were all below 5 (range: 1.023–2.145), and tolerance values were all above 0.4, indicating no significant multicollinearity.

The separate multivariable logistic regression models were then performed (**Table 5**). In Model 1, T MTV remained an independent risk factor for recurrence and metastasis (OR = 1.150, 95% CI: 1.052–1.257, P = 0.002). In Model 2, T TLG was independently associated with recurrence (OR = 1.035, 95% CI: 1.013–1.058, P = 0.002). In Model 3, N SUVmax remained significant (OR = 1.700, 95% CI: 1.050–2.752, P = 0.031). In Model 4, WB MTV was associated with recurrence (OR = 1.028, 95% CI: 0.960–1.100, P = 0.028). In Model 5, WB TLG remained an independent risk factor (OR = 1.028, 95% CI: 1.008–1.049, P = 0.006). LVI was significantly associated with recurrence in Models 1, 2, 3, and 5 (OR range: 2.512–2.987, all P < 0.05), but not in Model 4 (OR = 1.987, 95% CI: 0.876–4.508, P = 0.101). Pathological N stage was not independently associated with recurrence in any model (OR range: 1.423–1.689, all P > 0.05).

**Table 5:** Separate multivariable logistic regression models for recurrence and metastasis.

Model	Parameter	OR	95% CI	P
Model 1	T MTV	1.150	1.052–1.257	0.002
	LVI positive	2.875	1.023–8.082	0.045
	pN1	1.523	0.712–3.258	0.276
Model 2	T TLG	1.035	1.013–1.058	0.002
	LVI positive	2.764	0.987–7.741	0.053
	pN1	1.489	0.698–3.177	0.304
Model 3	N SUVmax	1.700	1.050–2.752	0.031
	LVI positive	2.512	0.912–6.918	0.074
	pN1	1.423	0.668–3.032	0.359
Model 4	WB MTV	1.028	0.960–1.100	0.028
	LVI positive	1.987	0.876–4.508	0.101
	pN1	1.689	0.785–3.634	0.180
Model 5	WB TLG	1.028	1.008–1.049	0.006
	LVI positive	2.987	1.078–8.276	0.035
	pN1	1.534	0.718–3.277	0.268

OR, odds ratio; CI, confidence interval; LVI, lymphovascular invasion; T, primary tumor; N, axillary lymph nodes; WB, whole body; SUV, standardized uptake value; MTV, metabolic tumor volume; TLG, total lesion glycolysis; pN, pathological N stage.

The ORs for PET parameters reported in our multivariable models represent the change in odds per one-unit increase. Because the units of MTV (cm³) and TLG (g) are large, the per-unit effect sizes appear modest. When recalculated for clinically meaningful increments, however, the associations become substantial (**Table 6**). A 10 cm³ increase in T MTV was associated with a four-fold increase in the odds of recurrence (OR = 4.046, 95% CI: 1.657–9.875, P = 0.002), and a 30 g increase in T TLG was associated with an approximately 2.8-fold increase (OR = 2.806, 95% CI: 1.492–5.276, P = 0.002).

**Table 6:** ORs for clinically meaningful increments in PET/CT parameters.

Parameter	Unit	OR per unit	Clinically relevant increment	OR for increment	95% CI	P
T MTV	1 cm^3^	1.150	10 cm^3^	4.046	1.657–9.875	0.002
T TLG	1 g	1.035	30 g	2.806	1.492–5.276	0.002
N SUVmax	1 unit	1.700	1 unit	1.700	1.050–2.752	0.031
WB MTV	1 cm^3^	1.028	10 cm^3^	1.319	0.665–2.613	0.028
WB TLG	1 g	1.028	30 g	2.290	1.257-4.172	0.006

OR, odds ratio; CI, confidence interval; T, primary tumor; N, axillary lymph nodes; WB, whole body; SUV, standardized uptake value; MTV, metabolic tumor volume; TLG, total lesion glycolysis.

### 3.6 ROC analysis

ROC curve analysis was performed to determine the optimal cut-off values for each PET/CT parameter (**Table 7**). T MTV demonstrated an AUC of 0.672, with an optimal cut-off of 23.755 cm^3^ (sensitivity 40.8%, specificity 87.7%). T TLG had an AUC of 0.612, with an optimal cut-off of 117.83 g (sensitivity 32.7%, specificity 94.7%). N SUVmax showed an AUC of 0.631, with an optimal cut-off of 3.245 (sensitivity 65.3%, specificity 61.4%). WB MTV exhibited an AUC of 0.638, with an optimal cut-off of 28.06 cm³ (sensitivity 61.2%, specificity 61.4%). WB TLG had an AUC of 0.650, with an optimal cut-off of 127.48 g (sensitivity 40.8%, specificity 84.2%). The combined ROC analysis yielded an AUC of 0.805, demonstrating improved predictive performance when these parameters were used in combination (**Figure 5**).

**Table 7:** ROC analysis and optimal cut-off values for PET/CT parameters.

Parameter	AUC	Best cut-off	Sensitivity	Specificity	Youden index	F1 score
T MTV	0.672	23.755	0.408	0.877	0.285	0.526
T TLG	0.612	117.830	0.327	0.947	0.274	0.471
N SUVmax	0.631	3.245	0.653	0.614	0.267	0.621
WB MTV	0.638	28.060	0.612	0.614	0.226	0.594
WB TLG	0.650	127.480	0.408	0.842	0.250	0.513

AUC, area under the curve; T, primary tumor; N, axillary lymph nodes; WB, whole body; SUV, standardized uptake value; MTV, metabolic tumor volume; TLG, total lesion glycolysis.

**Figure 5:**
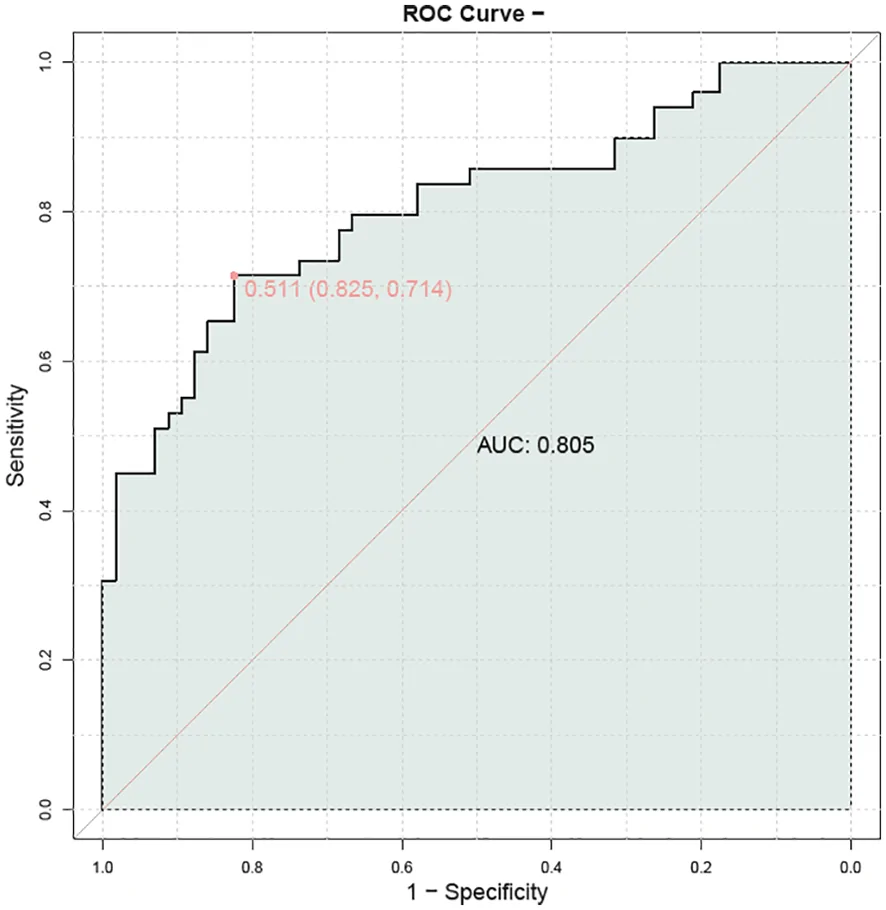
ROC curve of postoperative recurrence and metastasis in early breast cancer patients predicted by preoperative [18F]FDG PET/CT imaging parameters.

ROC, receiver operating characteristic; [18F]FDG PET/CT, 2-deoxy-2-[¹^8^F]fluoro-D-glucose positron emission tomography/computed tomography.

### 3.7 Subgroup analysis

Subgroup analyses were performed to evaluate the prognostic value of PET/CT metabolic parameters according to histologic type and type of surgery (**Tables 8**, **9**). Among patients with IDC (n = 85), all five PET/CT parameters remained significantly associated with recurrence (all P < 0.05). The number of patients with other histologic types (n = 21) was insufficient for stable multivariate estimation, and these cases were analyzed descriptively only. Among patients who underwent BCS (n = 44), T MTV, T TLG, N SUVmax, and WB TLG remained significantly associated with recurrence (all P < 0.05). Among patients who underwent mastectomy (n = 62), similar associations were observed for all four parameters (all P < 0.05). The interaction P values for all parameters were > 0.05, suggesting that the prognostic value of PET/CT metabolic parameters was consistent across histologic type and surgical modality strata.

**Table 8:** Subgroup analysis of PET/CT metabolic parameters for predicting recurrence by histologic type and surgery type.

Parameter	IDC (n=85)		Other type (n=21)		P for interaction
	OR (95% CI)	P	OR (95% CI)	P	
T MTV	1.142 (1.045–1.249)	0.003	–	–	0.847
T TLG	1.031 (1.010–1.053)	0.004	–	–	0.913
N SUVmax	1.648 (1.002–2.710)	0.039	–	–	0.762
WB MTV	1.023 (0.955–1.097)	0.033	–	–	0.891
WB TLG	1.025 (1.005–1.045)	0.015	–	–	0.805

IDC, invasive ductal carcinoma; OR, odds ratio; CI, confidence interval; T, primary tumor; N, axillary lymph nodes; WB, whole body; SUV, standardized uptake value; MTV, metabolic tumor volume; TLG, total lesion glycolysis.

**Table 9:** Subgroup analysis of PET/CT metabolic parameters for predicting recurrence by surgery type.

Parameter	BCS (n=44)		Mastectomy (n=62)		P for interaction
	OR (95% CI)	P	OR (95% CI)	P	
T MTV	1.128 (1.018–1.251)	0.021	1.156 (1.038–1.287)	0.008	0.724
T TLG	1.025 (1.003–1.048)	0.026	1.038 (1.012–1.065)	0.004	0.618
N SUVmax	1.512 (1.001–2.285)	0.049	1.782 (1.089–2.916)	0.021	0.592
WB TLG	1.019 (1.001–1.038)	0.041	1.032 (1.008–1.057)	0.009	0.531

BCS, breast-conserving surgery; OR, odds ratio; CI, confidence interval; T, primary tumor; N, axillary lymph nodes; WB, whole body; SUV, standardized uptake value; TLG, total lesion glycolysis.

## 4 Discussion

This study aimed to evaluate the predictive utility of preoperative [18F]FDG PET/CT imaging parameters for postoperative recurrence and metastasis in breast cancer patients. Our findings indicate that several imaging parameters, including T MTV, T TLG, N SUVmax, WB MTV, and WB TLG, are linked to a higher risk of recurrence and metastasis. Additionally, we observed significant differences in histological markers such as Ki-67 expression, ER positivity, PR positivity, and HER2 status between patients who experienced recurrence or metastasis and those who did not. These observations provide valuable understanding of the mechanisms driving tumor behavior and potential biomarkers for predicting disease progression.

LVI was more prevalent in the group that experienced recurrence or metastasis, suggesting its role as a prognostic factor. This finding aligns with prior studied by Wei et al. and Lee et al. (19, 20) indicating that LVI is a critical determinant of metastatic spread in breast cancer. The presence of LVI indicates that cancer cells have invaded the lymphatic system, facilitating their dissemination to distant sites (21). Mechanistically, this could be due to the disruption of normal tissue architecture and the activation of angiogenic pathways, which promote tumor cell migration and survival in circulation (22, 23).

Ki-67 expression, a marker of cellular proliferation, was found to be lower in the non-recurrence group than in the recurrence/metastasis group. Ki-67 is known to correlate with tumor aggressiveness and poor prognosis. Higher Ki-67 levels suggest increased proliferative activity, which may contribute to faster tumor growth and higher chances of metastasis. Previous research supports this observation, showing that high Ki-67 expression is linked to adverse outcomes in breast cancer patients. The biological mechanism behind this association likely involves enhanced DNA replication and cell cycle progression, leading to greater tumor burden and resistance to therapy (8, 24, 25).

ER and PR positivity were also more common in the non-recurrence group. Hormone receptor-positive tumors tend to grow more slowly and respond better to endocrine therapies, which can explain the reduced risk of recurrence in these patients (26, 27). ER and PR positivity influence tumor biology by modulating cell growth and differentiation through hormone-dependent signaling pathways. In contrast, the absence of these receptors may lead to uncontrolled cell proliferation and increased metastatic potential (28, 29).

HER2 positivity was more frequent in the recurrence/metastasis group, indicating its association with aggressive tumor behavior. HER2 overexpression activates multiple downstream signaling pathways that play roles in cell proliferation, survival, and migration. Consequently, HER2-positive tumors are often characterized by rapid growth and early metastasis. Targeted therapies against HER2 have improved outcomes, but the intrinsic aggressiveness of these tumors remains a challenge. Our findings reinforce the idea that HER2 status is a crucial determinant of disease progression and should be considered in treatment planning (30–32).

TNBC was more frequent in the recurrence/metastasis group, consistent with its aggressive phenotype. Published studies have shown that TNBC exhibits the highest FDG uptake among all molecular subtypes, with all metabolic parameters significantly associated with triple-negative status (33). In operable TNBC, lymph node metabolic and volumetric parameters are significant predictors of recurrence (34). These findings suggest that the prognostic information carried by TNBC is substantially reflected in the PET/CT metabolic parameters analyzed in this study.

Among the preoperative [18F]FDG PET/CT imaging parameters, T MTV and T TLG were higher in the recurrence/metastasis group. T MTV reflects the metabolic tumor volume, while T TLG combines both metabolic activity and tumor size. Elevated T MTV and T TLG suggest increased tumor burden and metabolic activity, which are indicative of aggressive tumor behavior. The biological rationale behind this observation lies in the Warburg effect, where cancer cells exhibit enhanced glucose metabolism to meet their energy demands. Increased metabolic activity can fuel tumor growth and facilitate metastatic spread by providing necessary substrates for cellular processes (35, 36).

N SUVmax was also higher in the recurrence/metastasis group. This parameter is indicative of increased metabolic activity in lymph nodes, potentially reflecting the presence of metastatic deposits. Elevated N SUVmax could be attributed to the upregulation of glucose transporters and increased glycolytic activity in metastatic cells. Such metabolic reprogramming enables cancer cells to survive and proliferate in the nutrient-poor environment of lymph nodes, thereby enhancing their metastatic potential (37, 38).

WB MTV and WB TLG were also higher in the recurrence/metastasis group. These metrics offer a comprehensive evaluation of systemic tumor load and metabolic activity. Elevated WB MTV and WB TLG suggest widespread disease involvement and increased likelihood of metastasis. The underlying mechanism likely involves systemic alterations in metabolism and immune evasion strategies employed by cancer cells to establish metastases at distant sites (35, 39).

In the multivariable analyses, we constructed separate models for each PET parameter rather than a single comprehensive model, because MTV and TLG are mathematically related and strongly correlated, and because the limited number of events precluded the inclusion of multiple correlated parameters. This approach avoided multicollinearity and maintained an adequate events-per-variable ratio (approximately 16.3 events per variable). T MTV, T TLG, N SUVmax, WB MTV, and WB TLG each remained independently associated with recurrence in their respective models, after adjusting for LVI and pathological N stage. These findings indicate that preoperative PET/CT metabolic parameters provide prognostic information that is independent of conventional clinicopathological factors. Notably, pathological N stage was not independently associated with recurrence in any model, which is consistent with the observed lack of significant difference in N stage distribution between the groups. LVI remained a significant risk factor in most models, consistent with its established prognostic role.

The incremental prognostic value of PET/CT parameters is a central finding of our study. In multivariable analyses, PET/CT metabolic parameters remained independently associated with recurrence after adjusting for LVI and pN, both of which are established prognostic factors in breast cancer. The effect sizes for PET/CT parameters, when expressed for clinically meaningful increments, are substantial—a 10 cm³ increase in T MTV was associated with a four-fold increase in the odds of recurrence (OR = 4.046, 95% CI: 1.661–9.748, P = 0.002). Previous studies have similarly demonstrated the incremental value of PET/CT parameters. A prediction model incorporating baseline [18F]FDG PET/CT metabolic parameters achieved a C-index of 0.907 for 5-year recurrence-free survival, demonstrating improved prognostic stratification beyond clinicopathological factors alone (40). The multi-omics prognostic stratification (MOPS) model integrating clinical data, FDG PET-CT, and medical reports outperformed single-omics models, TN staging, and molecular subtyping. From a biological perspective, MTV and TLG capture metabolic tumor burden, which is distinct from anatomical staging and reflects the Warburg effect—enhanced glucose metabolism in cancer cells (41). N SUVmax reflects the metabolic activity of axillary lymph nodes, which may capture metastatic potential beyond pathological nodal involvement. Clinically, preoperative PET/CT parameters could refine risk stratification beyond traditional factors, potentially identifying high-risk patients who might benefit from intensified surveillance or treatment escalation. The metabolic parameters identified in our study could also serve as candidate features for future multimodal models incorporating radiomics or multi-omics data.

Our combined ROC model achieved an AUC of 0.805, which is comparable to or exceeds that of several previously reported PET/CT-based models. For example, Nakajima et al. reported AUCs of 0.85 for MTV and 0.86 for TLG in postmastectomy patients with 1–3 positive axillary nodes (42). However, our AUC is lower than those reported for radiomics and deep learning models, which achieved AUCs of 0.91–0.99, albeit with larger sample sizes, more complex computational pipelines, and external validation (43). Several factors may explain these differences. First, our cohort consisted exclusively of patients with stage I–II disease who underwent upfront surgery, excluding patients receiving neoadjuvant therapy; this may have compressed the range of PET/CT parameter values and attenuated effect sizes. Second, our endpoint was recurrence within 24 months, which captures early recurrence but may not fully reflect long-term prognosis. Third, differences in PET/CT acquisition and analysis protocols across studies may affect parameter quantification. Finally, our statistical modeling approach—separate multivariable models for each PET parameter rather than a single comprehensive model—may yield more conservative performance estimates. Despite these differences, our model offers the advantage of clinical accessibility and interpretability, as it relies on routinely available metabolic parameters without requiring radiomic feature extraction or deep learning infrastructure.

The prognostic impact of histologic type and treatment modality warrants specific discussion. In our cohort, histologic type did not differ significantly between the occurred and non-occurred groups, and subgroup analyses demonstrated that PET/CT metabolic parameters retained their prognostic value among patients with IDC. This is consistent with published evidence demonstrating that histologic type (ductal vs. lobular) is not an independent prognostic factor for disease-free survival (DFS) (44, 45). Regarding surgical modality, BCS and mastectomy were equally distributed between the two groups, and published studies have consistently demonstrated equivalent survival outcomes between these two surgical approaches in early-stage breast cancer (46). Notably, among patients with TNBC and stage I disease, BCS has been associated with a lower local-regional recurrence rate than mastectomy (relative risk [RR] 0.5, P = 0.02) (46). Our subgroup analyses demonstrated that the prognostic value of PET/CT metabolic parameters was consistent across histologic type and surgical modality strata, suggesting that these imaging biomarkers provide prognostic information that is independent of these pathological and surgical factors. Additionally, published evidence indicates that IDC tends to demonstrate higher metabolic activity on [18F]FDG PET/CT compared with invasive lobular carcinoma, which often exhibits lower FDG uptake due to its histological characteristics (47). This differential metabolic behavior may partially explain why PET/CT parameters performed consistently across histologic types in our cohort.

The prognostic value of preoperative PET/CT metabolic parameters should also be interpreted in the context of adjuvant treatment. Although our retrospective study did not systematically collect detailed adjuvant treatment information, published evidence consistently demonstrates that baseline metabolic parameters provide prognostic information independent of subsequent adjuvant therapy. Park et al. demonstrated that SUV peak at baseline PET/CT was the only factor independently associated with DFS in pathologic stage II IDC, showing a stronger association with DFS than pathologic prognostic stage or TNM stage (48). Importantly, although higher metabolic parameters were associated with poorer DFS, patients with higher SUV peak or tumor-to-liver ratio (TLR) values derived greater benefit from adjuvant chemotherapy, indicating that metabolic parameters identify tumors with higher biological aggressiveness that are more chemo sensitive. Cha et al. further demonstrated that in operable breast cancer patients without neoadjuvant chemotherapy, primary tumor SUVmax remained the only independent prognostic factor for recurrence in multivariate analysis that included adjuvant therapy status (49). These findings suggest that the association between preoperative PET/CT parameters and recurrence reflects the intrinsic biological behavior of the tumor rather than being confounded by treatment effects.

Several limitations of our model warrant acknowledgment. First, this was a retrospective, single-center study, which limits the generalizability of our findings; prognostic models often exhibit reduced performance in external validation cohorts. Second, the relatively small sample size and limited number of events constrained the number of variables that could be included in any single multivariable model, although we maintained an adequate events-per-variable ratio of approximately 16.3 by constructing separate models for each PET parameter. Third, the model was developed and evaluated using data from the same cohort, without independent external validation. Fourth, the 24-month follow-up period captures early recurrence but does not cover the second recurrence peak at 5–6 years. Fifth, detailed information on specific adjuvant treatment regimens was not systematically collected, introducing potential unmeasured confounding. Sixth, the quantification of nodal PET/CT parameters in patients without detectable FDG-avid lymph nodes relied on a standardized background ROI approach, which may introduce measurement variability. Seventh, the absence of genomic and multi-omics data is a significant limitation, as these data could have offered additional predictive utility. Finally, our cohort consisted exclusively of patients with stage I–II disease who underwent upfront surgery without neoadjuvant therapy, which may limit the applicability of our findings to the broader early-stage breast cancer population.

In summary, our research demonstrates that preoperative [18F]FDG PET/CT imaging metrics, particularly T MTV, T TLG, N SUVmax, WB MTV, and WB TLG, are linked to an increased risk of recurrence and metastasis in breast cancer patients. These results underscore the significance of metabolic imaging in evaluating tumor aggressiveness and directing personalized treatment approaches. Further research is warranted to validate these observations and investigate their clinical implications, paving the way for more effective management of breast cancer.

## 5 Conclusion

Preoperative [18F]FDG PET/CT imaging metrics, including T MTV, T TLG, N SUVmax, WB MTV, and WB TLG, may serve as potential predictors of early postoperative recurrence and metastasis within 24 months in early-stage breast cancer patients. These metabolic parameters reflect tumor burden and metabolic activity, which may influence early disease progression. Prospective studies with extended follow-up are necessary to validate these findings and to assess the whether these parameters also predict long-term outcomes.

## Data Availability

The raw data supporting the conclusions of this article will be made available by the authors, without undue reservation.

## References

[1] Sung H Filho AM Laversanne M Ferlay J Siegel RL Soerjomataram I . Global cancer statistics 2024: GLOBOCAN estimates of incidence and mortality worldwide for 34 cancers in 186 countries. CA: A Cancer J For Clin. (2026) 76:e70090. doi: 10.3322/caac.70090 42417444 PMC13343830

[2] Sinberger LA Zahavi T Keren-Khadmy N Dugach Y Sonnenblick A Salmon-Divon M . Refining prognostic tools for luminal breast cancer: genetic insights and comprehensive analysis. ESMO Open. (2025) 10:105080. doi: 10.1016/j.esmoop.2025.105080 40305907 PMC12088756

[3] Gennari A André F Barrios CH Cortés J de Azambuja E DeMichele A . ESMO Clinical Practice Guideline for the diagnosis, staging and treatment of patients with metastatic breast cancer. Ann Oncol Off J Eur Soc For Med Oncol. (2021) 32:1475–95. doi: 10.1016/j.annonc.2021.09.019 34678411

[4] Taralli S Orlandi A Pafundi PC Tempesta V Di Leone A Pontolillo L . Baseline (18)F-FDG PET/CT for predicting pathological response to neoadjuvant chemotherapy and prognosis in locally advanced breast cancer patients: analysis of tumor and lymphoid organs metabolic parameters. La Radiologia Med. (2025) 130:422–37. doi: 10.1007/s11547-025-01961-9 39937369

[5] Najid S Seban RD Champion L De Moura A Sebbag C Salaün H . Clinical utility of pre-therapeutic [18F]FDG PET/CT imaging for predicting outcomes in breast cancer. J Clin Med. (2023) 12:5487. doi: 10.3390/jcm12175487 37685551 PMC10488013

[6] Iqbal R Yaqub M Bektas HO Oprea-Lager DE de Vries EGE Glaudemans A . 18F]FDG and [18F]FES PET/CT imaging as a biomarker for therapy effect in patients with metastatic ER+ breast cancer undergoing treatment with Rintodestrant. Clin Cancer Research: Off J Am Assoc For Cancer Res. (2023) 29:2075–84. doi: 10.1158/1078-0432.CCR-22-2720 36735488

[7] Tello Galán MJ García Vicente AM Pérez Beteta J Amo Salas M Jiménez Londoño GA Pena Pardo FJ . Global heterogeneity assessed with (18)F-FDG PET/CT. Relation with biological variables and prognosis in locally advanced breast cancer. Rev Esp Med Nucl Imagen Mol (Engl Ed). (2019) 38:290–7. doi: 10.1016/j.remn.2019.02.004 31427247

[8] Martins-Branco D Nader-Marta G Molinelli C Ameye L Paesmans M Ignatiadis M . Ki-67 index after neoadjuvant endocrine therapy as a prognostic biomarker in patients with ER-positive/HER2-negative early breast cancer: a systematic review and meta-analysis. Eur J Cancer (Oxford England: 1990). (2023) 194:113358. doi: 10.1016/j.ejca.2023.113358 37857118

[9] Gao Y Yin L Ma L Wu C Zhu X Liu H . Comparative analysis of metabolic characteristics and prognostic stratification of HER2-low and HER2-zero breast cancer using (18)F-FDG PET/CT imaging. Cancer Imaging: Off Publ Int Cancer Imaging Soc. (2024) 24:166. doi: 10.1186/s40644-024-00812-6 39695831 PMC11653929

[10] Kitano Y Miyake KK Nobashi TW Ishimori T Nakamoto R Koyasu S . Subcutaneous/cutaneous uptake in the cancer-affected breast on (18)F-FDG PET/CT: association with post-surgical prognosis in breast cancer patients. Ann Nucl Med. (2026) 40:73–86. doi: 10.1007/s12149-025-021106-8 41046498

[11] Evangelista L Urso L Caracciolo M Stracuzzi F Panareo S Cistaro A . FDG PET/CT volume-based quantitative data and survival analysis in breast cancer patients: a systematic review of the literature. Curr Med Imaging. (2023) 19:807–16. doi: 10.2174/1573405618666220329094423 35352652

[12] Groheux D Martineau A Teixeira L Espié M de Cremoux P Bertheau P . (18)FDG-PET/CT for predicting the outcome in ER+/HER2- breast cancer patients: comparison of clinicopathological parameters and PET image-derived indices including tumor texture analysis. Breast Cancer Research: BCR. (2017) 19:3. doi: 10.1186/s13058-016-0793-2 28057031 PMC5217422

[13] Fan L Strasser-Weippl K Li JJ St Louis J Finkelstein DM Yu KD . Breast cancer in China. Lancet Oncol. (2014) 15:e279–89. doi: 10.1016/s1470-2045(13)70567-9 24872111

[14] Amin MB Greene FL Edge SB Compton CC Gershenwald JE Brookland RK . The Eighth Edition AJCC Cancer Staging Manual: Continuing to build a bridge from a population-based to a more "personalized" approach to cancer staging. CA: A Cancer J For Clin. (2017) 67:93–9. doi: 10.3322/caac.21388 28094848

[15] Cardoso F Harbeck N Fallowfield L Kyriakides S Senkus E . Locally recurrent or metastatic breast cancer: ESMO Clinical Practice Guidelines for diagnosis, treatment and follow-up. Ann Oncol Off J Eur Soc For Med Oncol. (2012) 23(7):vii11–9. doi: 10.1093/annonc/mds232 22997442

[16] Allred DC Harvey JM Berardo M Clark GM . Prognostic and predictive factors in breast cancer by immunohistochemical analysis. Modern Pathology: Off J United States Can Acad Pathology Inc. (1998) 11:155–68. 9504686

[17] Moeder CB Giltnane JM Harigopal M Molinaro A Robinson A Gelmon K . Quantitative justification of the change from 10% to 30% for human epidermal growth factor receptor 2 scoring in the American Society of Clinical Oncology/College of American Pathologists guidelines: tumor heterogeneity in breast cancer and its implications for tissue microarray based assessment of outcome. J Clin Oncol Off J Am Soc Clin Oncol. (2007) 25:5418–25. doi: 10.1200/jco.2007.12.8033 18048824

[18] Goldhirsch A Winer EP Coates AS Gelber RD Piccart-Gebhart M Thürlimann B . Personalizing the treatment of women with early breast cancer: highlights of the St Gallen International Expert Consensus on the Primary Therapy of Early Breast Cancer 2013. Ann Oncol Off J Eur Soc For Med Oncol. (2013) 24:2206–23. doi: 10.1093/annonc/mdt303 23917950 PMC3755334

[19] Wei C Deng Y Wei S Huang Z Xie Y Xu J . Lymphovascular invasion is a significant risk factor for non-sentinel nodal metastasis in breast cancer patients with sentinel lymph node (SLN)-positive breast cancer: a cross-sectional study. World J Surg Oncol. (2023) 21:386. doi: 10.1186/s12957-023-03273-6 38097994 PMC10720167

[20] Lee SJ Go J Ahn BS Ahn JH Kim JY Park HS . Lymphovascular invasion is an independent prognostic factor in breast cancer irrespective of axillary node metastasis and molecular subtypes. Front Oncol. (2023) 13:1269971. doi: 10.3389/fonc.2023.1269971 38053656 PMC10694501

[21] Kuhn E Gambini D Despini L Asnaghi D Runza L Ferrero S . Updates on lymphovascular invasion in breast cancer. Biomedicines. (2023) 11:968. doi: 10.3390/biomedicines11030968 36979946 PMC10046167

[22] Zhang C Zhou P Li R Li Z Ouyang A . Prediction of lymphovascular invasion in invasive breast cancer based on clinical-MRI radiomics features. BMC Med Imaging. (2024) 24:277. doi: 10.1186/s12880-024-01456-5 39415127 PMC11481431

[23] Null JL Kim DJ McCann JV Pramoonjago P Fox JW Zeng J . Periostin+ stromal cells guide lymphovascular invasion by cancer cells. Cancer Res. (2023) 83:2105–22. doi: 10.1158/0008-5472.CAN-22-2412 37205636 PMC10330490

[24] Torlakovic EE Baniak N Barnes PJ Chancey K Chen L Cheung C . Fit-for-purpose Ki-67 immunohistochemistry assays for breast cancer. Lab Investigation; A J Tech Methods Pathol. (2024) 104:102076. doi: 10.1016/j.labinv.2024.102076 38729353

[25] Tabnak P HajiEsmailPoor Z Baradaran B Pashazadeh F Aghebati Maleki L . MRI-based radiomics methods for predicting Ki-67 expression in breast cancer: a systematic review and meta-analysis. Acad Radiol. (2024) 31:763–87. doi: 10.1016/j.acra.2023.10.010 37925343

[26] Diep CH Spartz A Truong TH Dwyer AR El-Ashry D Lange CA . Progesterone receptor signaling promotes cancer associated fibroblast mediated tumorigenicity in ER+ breast cancer. Endocrinology. (2024) 165:bqae092. doi: 10.1210/endocr/bqae092 39041201 PMC11492492

[27] Choong GM Hoskin TL Boughey JC Ingle JN Goetz MP . Endocrine therapy omission in estrogen receptor-low (1%-10%) early-stage breast cancer. J Clin Oncol Off J Am Soc Clin Oncol. (2025) 43:1875–85. doi: 10.1200/jco-24-02263 40215443 PMC12119225

[28] Hashmi AA Mallick BA Rashid K Malik UA Zia S Zia F . Significance of estrogen/progesterone receptor expression in metaplastic breast carcinoma. Dis Markers. (2024) 2024:2540356. doi: 10.1155/2024/2540356 38601434 PMC11006451

[29] Dou H Li F Wang Y Chen X Yu P Jia S . Estrogen receptor-negative/progesterone receptor-positive breast cancer has distinct characteristics and pathologic complete response rate after neoadjuvant chemotherapy. Diagn Pathol. (2024) 19:5. doi: 10.1186/s13000-023-01433-6 38178166 PMC10765627

[30] Wolff AC Somerfield MR Dowsett M Hammond MEH Hayes DF McShane LM . Human epidermal growth factor receptor 2 testing in breast cancer. Arch Pathol Lab Med. (2023) 147:993–1000. doi: 10.5858/arpa.2023-0950-SA 37303228

[31] Wolff AC Somerfield MR Dowsett M Hammond MEH Hayes DF McShane LM . Human epidermal growth factor receptor 2 testing in breast cancer: ASCO-College of American Pathologists guideline update. J Clin Oncol Off J Am Soc Clin Oncol. (2023) 41:3867–72. doi: 10.1200/jco.22.02864 37284804

[32] Huppert LA Gumusay O Idossa D Rugo HS . Systemic therapy for hormone receptor-positive/human epidermal growth factor receptor 2-negative early stage and metastatic breast cancer. CA: A Cancer J For Clin. (2023) 73:480–515. doi: 10.3322/caac.21777 36939293

[33] Kajáry K Tőkés T Dank M Kulka J Szakáll S Jr Lengyel Z . Correlation of the value of 18F-FDG uptake, described by SUVmax, SUVavg, metabolic tumour volume and total lesion glycolysis, to clinicopathological prognostic factors and biological subtypes in breast cancer. Nucl Med Commun. (2015) 36:28–37. doi: 10.1097/MNM.0000000000000217 25299471

[34] Kaida H Toh U Hayakawa M Hattori S Fujii T Kurata S . The relationship between 18F-FDG metabolic volumetric parameters and clinicopathological factors of breast cancer. Nucl Med Commun. (2013) 34:562–70. doi: 10.1097/MNM.0b013e328360d945 23619341

[35] Tokac RH Tokac ECB Yararbas U Aykut A Durmaz A Akin H . Cross-sectional analysis of metabolic tumor burden detected by F-18 FDG PET/CT and circulating tumor DNA in advanced breast cancer. Cancer Med. (2025) 14:e71049. doi: 10.1002/cam4.71049 40824151 PMC12359722

[36] Tamam M Ozcevik H Kulduk G Acar Tayyar MN Babacan GB . Evaluating the correlation between pretreatment (18)F-FDG PET/CT metabolic parameters and tumor-infiltrating lymphocyte levels in nonluminal breast cancer and impact on survival. Pathol Oncol Research: POR. (2024) 30:1612014. doi: 10.3389/pore.2024.1612014 39839836 PMC11750436

[37] Shin DJ Choi H Oh DK Sung HP Kim JH Kim DH . Correlation between standardized uptake value of (18)F-FDG PET/CT and conductivity with pathologic prognostic factors in breast cancer. Sci Rep. (2023) 13:9844. doi: 10.1038/s41598-023-36958-9 37330544 PMC10276807

[38] Erol M Önner H Karanis MIE Şen AE . Quantitative assessment of volume-based metabolic parameters in early-stage invasive ductal breast cancer: impact of different standardized uptake value thresholds on prognostic factors - a comprehensive analysis. Hellenic J Nucl Med. (2024) 27:229–35. doi: 10.1967/s002449912758 39644275

[39] Ozawa H Higuchi T Fujimoto Y Bun A Fukui R Kanaoka H . Total lesion glycolysis levels as predictive indicators in patients with metastatic and recurrent breast cancer undergoing endocrine therapy with or without CDK4/6 inhibitor. Anticancer Res. (2022) 42:4813–24. doi: 10.21873/anticanres.15986 36191999

[40] Tatoğlu MT Ökten IN İbişoğlu E Söylemez Akkurt T Tozan E Tatoğlu FY . Integrating PET/CT-derived heterogeneity indices and composite risk scores improves prognostic stratification in breast cancer. Eur J Nucl Med Mol Imaging. (2026) 53:2439–54. doi: 10.1007/s00259-025-07622-3 41191096

[41] Liang X Zhang T Braga M Han L Donswijk M Huang J . Multi-omics deep learning improves FDG PET-CT-based long-term prognostication of breast cancer. NPJ Precis Oncol. (2026) 10:74. doi: 10.1038/s41698-026-01283-7 41611944 PMC12921030

[42] Nakajima N Kataoka M Sugawara Y Ochi T Kiyoto S Ohsumi S . Volume-based parameters of 18F-fluorodeoxyglucose positron emission tomography/computed tomography improve disease recurrence prediction in postmastectomy breast cancer patients with 1 to 3 positive axillary lymph nodes. Int J Radiat Oncol Biol Phys. (2013) 87:738–46. doi: 10.1016/j.ijrobp.2013.08.001 24064322

[43] Kawaji K Nakajo M Shinden Y Jinguji M Tani A Hirahara D . Application of machine learning analyses using clinical and [(18)F]-FDG-PET/CT radiomic characteristics to predict recurrence in patients with breast cancer. Mol Imaging Biol. (2023) 25:923–34. doi: 10.1007/s11307-023-01823-8 37193804

[44] Fritz P Klenk S Goletz S Gerteis A Simon W Brinkmann F . Clinical impacts of histological subtyping primary breast cancer. Anticancer Res. (2010) 30:5137–44. 21187502

[45] Kwast AB Groothuis-Oudshoorn KC Grandjean I Ho VK Voogd AC Menke-Pluymers MB . Histological type is not an independent prognostic factor for the risk pattern of breast cancer recurrences. Breast Cancer Res Treat. (2012) 135:271–80. doi: 10.1007/s10549-012-2160-z 22810087

[46] Yi M Chavez-MacGregor M Smith BD Mitchell MP Caudle AS DeSnyder SM . Comparing survival differences between patients undergoing breast-conserving therapy versus total mastectomy as a first treatment for early stage breast cancer. Ann Surg Oncol. (2025) 32:9757–66. doi: 10.1245/s10434-025-18030-0 40789993 PMC12352611

[47] Ge C Shi L Tan Z . (18)F-fluorodeoxyglucose positron emission tomography-computed tomography for predicting pathological complete response to neoadjuvant chemotherapeutic in breast cancer patients. Gland Surg. (2025) 14:48–59. doi: 10.21037/gs-2024-568 39958895 PMC11826249

[48] Park HL Lee SW Hong JH Lee J Lee A Kwon SJ . Prognostic impact of (18)F-FDG PET/CT in pathologic stage II invasive ductal carcinoma of the breast: re-illuminating the value of PET/CT in intermediate-risk breast cancer. Cancer Imaging: Off Publ Int Cancer Imaging Soc. (2023) 23:2. doi: 10.1186/s40644-022-00519-6 36600314 PMC9811771

[49] Cha J Park HS Kim D Kim HJ Kim MJ Cho YU . A hierarchical prognostic model for risk stratification in patients with early breast cancer according to (18) F-fludeoxyglucose uptake and clinicopathological parameters. Cancer Med. (2018) 7:1127–34. doi: 10.1002/cam4.1394 29479851 PMC5911607

